# Prolonged viral suppression with anti-HIV-1 antibody therapy

**DOI:** 10.1038/s41586-022-04597-1

**Published:** 2022-04-13

**Authors:** Christian Gaebler, Lilian Nogueira, Elina Stoffel, Thiago Y. Oliveira, Gaëlle Breton, Katrina G. Millard, Martina Turroja, Allison Butler, Victor Ramos, Michael S. Seaman, Jacqueline D. Reeves, Christos J. Petroupoulos, Irina Shimeliovich, Anna Gazumyan, Caroline S. Jiang, Nikolaus Jilg, Johannes F. Scheid, Rajesh Gandhi, Bruce D. Walker, Michael C. Sneller, Anthony Fauci, Tae-Wook Chun, Marina Caskey, Michel C. Nussenzweig

**Affiliations:** 1 Laboratory of Molecular Immunology, The Rockefeller University, New York, NY USA; 2 Columbia University Irving Medical Center, New York, NY USA; 3 Center for Virology and Vaccine Research, Beth Israel Deaconess Medical Center, Harvard Medical School, Boston, MA USA; 4 Labcorp-Monogram Biosciences, LabCorp, South San Francisco, CA USA; 5 Center for Clinical and Translational Science, The Rockefeller University, New York, NY USA; 6 Division of Infectious Diseases, Massachusetts General Hospital, Boston, MA USA; 7 Division of Gastroenterology, Massachusetts General Hospital, Boston, MA USA; 8 Ragon Institute of MGH, MIT and Harvard, Cambridge, MA USA; 9 Laboratory of Immunoregulation, National Institute of Allergy and Infectious Diseases (NIAID), National Institutes of Health (NIH), Bethesda, MD USA; 10 Howard Hughes Medical Institute, The Rockefeller University, New York, NY USA

**Keywords:** Retrovirus, Immunotherapy, Viral reservoirs

## Abstract

HIV-1 infection remains a public health problem with no cure. Anti-retroviral therapy (ART) is effective but requires lifelong drug administration owing to a stable reservoir of latent proviruses integrated into the genome of CD4^+^ T cells^1^. Immunotherapy with anti-HIV-1 antibodies has the potential to suppress infection and increase the rate of clearance of infected cells^2,3^. Here we report on a clinical study in which people living with HIV received seven doses of a combination of two broadly neutralizing antibodies over 20 weeks in the presence or absence of ART. Without pre-screening for antibody sensitivity, 76% (13 out of 17) of the volunteers maintained virologic suppression for at least 20 weeks off ART. Post hoc sensitivity analyses were not predictive of the time to viral rebound. Individuals in whom virus remained suppressed for more than 20 weeks showed rebound viraemia after one of the antibodies reached serum concentrations below 10 µg ml^−1^. Two of the individuals who received all seven antibody doses maintained suppression after one year. Reservoir analysis performed after six months of antibody therapy revealed changes in the size and composition of the intact proviral reservoir. By contrast, there was no measurable decrease in the defective reservoir in the same individuals. These data suggest that antibody administration affects the HIV-1 reservoir, but additional larger and longer studies will be required to define the precise effect of antibody immunotherapy on the reservoir.

## Abstract (web summary)

A clinical study shows that immunotherapy with anti-HIV-1 antibodies maintains prolonged viral suppression after anti-retroviral treatment is discontinued and affects the size and composition of the intact but not the defective proviral reservoir.

## Main

ART is highly effective in suppressing viral replication and preventing disease progression. However, HIV-1 infection persists in infected CD4^+^ T cells, which thus constitute a long-lived latent proviral reservoir^1^. Consequently, when ART is discontinued, viral rebound occurs within four weeks in most individuals^4^. So far, attempts to alter the latent viral reservoir and induce ART-free HIV-1 remission in humans have shown limited success^5,6^.

Nevertheless, studies in humanized mice and macaques show that monoclonal antibody administration can suppress HIV-1 or simian–human immunodeficiency virus (SHIV) infection for several years after therapy is terminated, suggesting that antibodies might have a role in therapies intended to achieve HIV-1 remission^7–9^. Antibodies may do so because they can neutralize the pathogen directly, have the potential to clear the virus and infected cells, and produce immune complexes that can enhance adaptive immunity^2^.

Administration of anti-HIV-1 broadly neutralizing antibodies (bNAbs) to humans is safe, and effective in preventing infection, lowering viraemia and maintaining viral suppression of antibody-sensitive viruses for short periods of time in the absence of ART^3,10–15^. Therefore, it has been suggested that broadly neutralizing antibodies could have a role as adjuncts to ART, or as a standalone maintenance treatment that aims to induce long-term ART-free HIV-1 remission^3^. Here we assessed the effectiveness of repeated bNAb combination therapy for the maintenance of long-term viral suppression as well as its effects on the size and composition of the latent viral reservoir in people living with HIV.

**Fig. 1:**
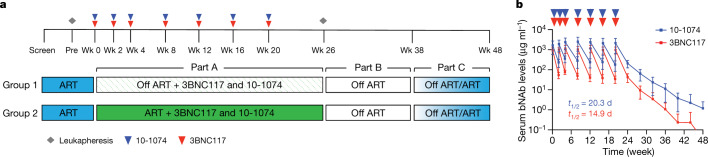
Study design and pharmacokinetics of 3BNC117 and 10-1074. **a**, Study design. Diamond represents time points of leukapheresis. Red and blue triangles represent 3BNC117 and 10-1074 infusions, respectively. Wk, week. **b**, Levels of 3BNC117 (red) and 10-1074 (blue) in serum (*n* = 23 participants), as determined by TZM-bl assay^18^. Data are mean ± s.d. Red and blue triangles indicate 3BNC117 and 10-1074 infusions, respectively. Mean half-life (*t*_1/2_) of each bNAb is indicated in days.

To study the safety, tolerability and anti-viral activity of the combination of two broad and potent anti-HIV-1 antibodies, 3BNC117 and 10-1074^16,17^ in the presence or absence of ART, we conducted a phase 1b, open-label, randomized clinical trial of two treatment groups of chronically infected people living with HIV (Fig. 1a). The primary objectives of the study were to evaluate the effects of repeated antibody infusions on maintaining viral suppression in the absence of ART, to assess effects on reservoir size when the antibodies were given alone or during suppressive ART, and to evaluate the safety of seven antibody infusions over five months.

**Extended Data Fig. 1:**
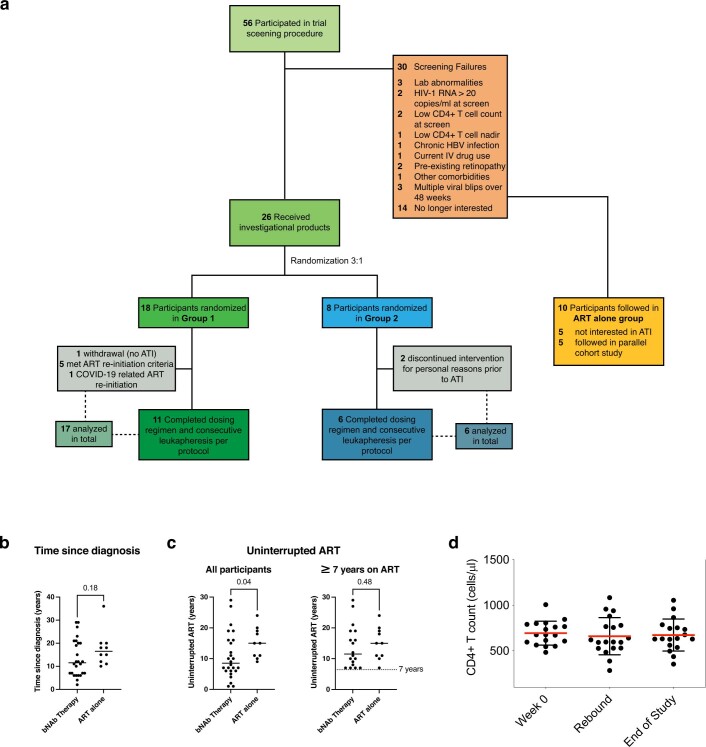
Study participant selection. **a**, Diagram depicts the selection of study participants. **b**, Comparison of time since HIV diagnosis between bNAb Therapy and ART-alone group. Statistical significance was determined using two-tailed Mann–Whitney U-test. Horizontal bars indicate median values **c**, Comparison of time of uninterrupted ART between bNAb Therapy and ART alone group for all participants (left panel) and all participants with uninterrupted ART for at least 7 years (right panel). Statistical significance was determined using two-tailed Mann–Whitney U-test. Horizontal bars indicate median values. **d**, Absolute CD4+ T cell counts at time of first antibody infusion (n = 18), time of viral rebound (n = 19) and at the end of the study (n = 18) (see also Supplementary Tables 4 and 5). Red lines indicate mean, error bars indicate standard deviation and individual participants are shown as dots.

Study eligibility criteria included ongoing ART and viral suppression with plasma HIV-1 RNA levels of less than 50 copies per ml for at least 12 months (with one blip of fewer than 500 copies per ml allowed), fewer than 20 copies per ml at screening, and CD4^+^ T cell counts above 500 cells per μl (Supplementary Table 1). Individuals whose regimens contained non-nucleoside reverse transcriptase inhibitors (NNRTIs) were switched to an integrase inhibitor-based regimen (dolutegravir plus tenofovir disoproxil fumarate and emtricitabine) four weeks before discontinuing ART owing to the long half-life of NNRTIs. The 26 participants who enrolled in the study were not screened for bNAb sensitivity of their proviral reservoir (Extended Data Fig. 1a, Extended Data Table 1a, Supplementary Table 1). Ten additional individuals on suppressive ART were followed over time in parallel under an observational study for repeated blood donations and reservoir assessments while remaining on suppressive ART (ART-alone group; Extended Data Fig. 1a, Extended Data Table 1b, Supplementary Table 2). The median time since diagnosis was 11.5 years and median time on uninterrupted suppressive ART was 8.5 years in the treatment groups and 16.5 and 15 years, respectively, in the ART-alone group (*P* = 0.18 and 0.04, respectively; Extended Data Fig. 1b, c, Extended Data Table 1a, b, Supplementary Tables 1, 2).

Participants in the treatment groups received up to seven infusions of 30 mg kg^−1^ of each antibody over the course of 20 weeks at 2-week (infusions 1–3) and 4-week intervals (infusions 4–7) (Fig. 1a). Eighteen of the 26 enrolled participants were randomized to group 1 and eight were randomized to group 2 (Extended Data Fig. 1a, Supplementary Table 1). Participants in group 1 were asked to discontinue their ART 2 days after the first 3BNC117 and 10-1074 infusions, whereas participants in group 2 remained on ART before discontinuing the antiretrovirals after 26 weeks (Fig. 1a). Viral load and CD4^+^ T cell counts were monitored on a weekly basis during analytical treatment interruption until week 38, every two weeks from weeks 38 until the end of follow-up at week 48, and every 4 to 8 weeks following ART re-initiation (see Methods).

All participants were followed for a total of 48 weeks from time of enrolment (day 0). Leukapheresis was performed approximately 2 weeks before and 26 weeks after the first antibody infusions in individuals who maintained viral loads below 20 copies per ml for at least 26 weeks.

Repeated antibody infusions over the course of 20 weeks were generally safe and well-tolerated. Eighty-eight per cent (79 out of 90) of the reported adverse events were of grade 1 severity—28% (25 out of 90) were considered at least possibly related to the antibodies and were of grade 1 severity. Two participants acquired hepatitis C virus (HCV) infection during study follow-up and experienced transient grade 3 elevations of hepatic transaminases, and ART was restarted. No serious adverse events were reported (Supplementary Table 3).

**Extended Data Fig. 2:**
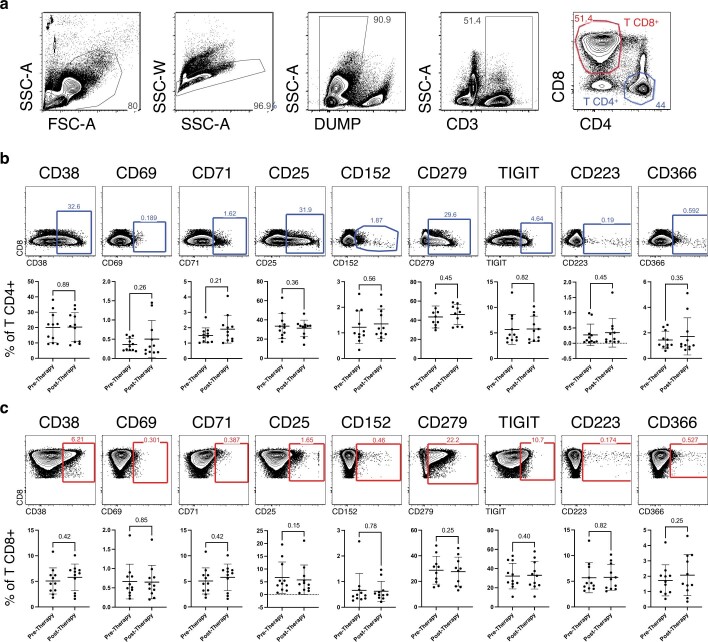
CD4^+^ and CD8^+^ T cell activation patterns pre- and post-Therapy. **a**, Gating strategy used to define total CD4^+^ (blue) and CD8^+^ (red) T cells. Dump channel includes CD19, CD20, CD14 and CD66b as well as Live/Dead. CD4+ T cells are identified as Dump−CD14−CD3+CD4+, whereas CD8+ T cells are Dump−CD14−CD3+CD8+. **b**, Representative flow cytometry plots and frequencies of CD4^+^ T cells expressing CD38, CD69, CD71, CD25, CD152, CD279, TIGIT, CD223 and CD366. **c**, Representative flow cytometry plots and frequencies of CD8^+^ T cells expressing CD38, CD69, CD71, CD25, CD152, CD279, TIGIT, CD223 and CD366. Relative change in frequencies was assessed between pre-therapy (T1) and 26-week post-therapy time points (T2). All panels represent the mean ± SD of 11 different donors. Significance was determined by two-tailed paired Student’s t-test.

The mean CD4^+^ T cell count was 729 (range 483–1,192) and 660 (range 284–1,083) cells per μl at the time of first antibody infusion and at rebound, respectively (Extended Data Fig. 1d, Supplementary Tables 4, 5). There were no significant changes in activation markers expressed by CD4^+^ or CD8^+^ T cells between the initial and 26-week time points (Extended Data Fig. 2a–c). Participant 5108 (group 1) experienced a more than 30% decline in absolute CD4^+^ T cell count from baseline (day 0) at week 18 despite maintaining undetectable plasma HIV-1 RNA. Per protocol, he did not receive the week 20 antibody infusions and was advised to resume ART. However, this participant chose to remain off ART until viral rebound, which occurred at week 24, when his CD4^+^ T cell was 640 cells per μl (Supplementary Table 4). In groups 1 and 2, re-initiation of ART after viral rebound resulted in control of viraemia to HIV-1 RNA at fewer than 50 copies per ml after 8 and 6 weeks, respectively (Supplementary Tables 4, 5). We conclude that repeated infusions of 3BNC117 and 10-1074 are generally safe and well-tolerated.

## Pharmacokinetics

**Extended Data Fig. 3:**
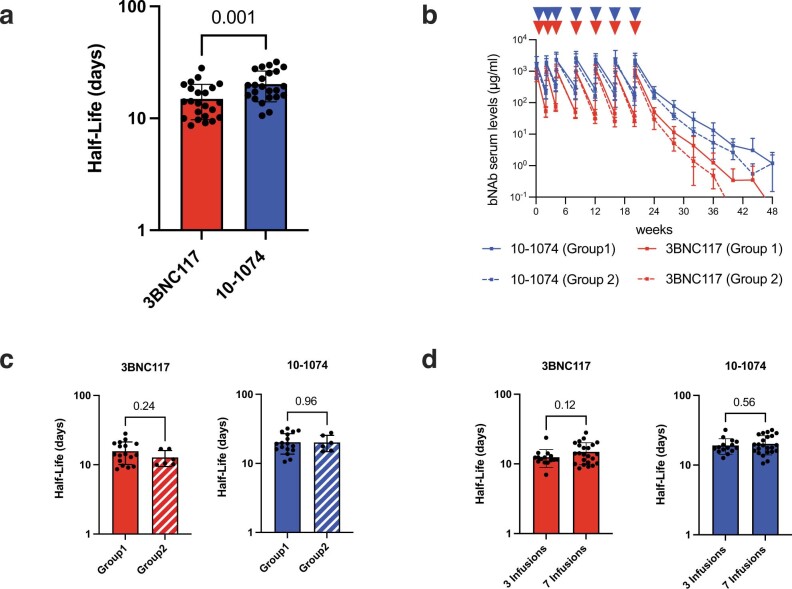
Pharmacokinetics of 3BNC117 and 10-1074. **a**, Scatter bar plots show half-lives of 3BNC117 (red) and 10–1074 (blue). Each dot represents a single participant (n = 23). Bars show mean values and standard deviation. Statistical significance was determined using a two-tailed paired t-test. **b**, 3BNC117 (red) and 10–1074 (blue) levels in serum for Group 1 (n = 17) and Group 2 (n = 6) participants. Curves indicate mean serum antibody concentrations and error bars represent standard deviation. **c**, Scatter bar plots show half-lives of 3BNC117 (red) and 10–1074 (blue) separated for Group 1 (n = 17) and Group 2 (n = 6) participants. Each dot represents a single participant. Bars show mean values and standard deviation. Statistical significance was determined using a two-tailed unpaired t-test. **d**, 3BNC117 (red) and 10-1074 (blue) half-live comparison between individuals receiving up to 3 infusions of the combination of 3BNC117 + 10–1074^12^ (n = 15) and participants receiving up to 7 infusions of the combination of 3BNC117 + 10–1074 (n = 23). Each dot represents a single participant. Bars show mean values and standard deviation. Statistical significance was determined using a two-tailed unpaired t-test.

Serum concentrations of 3BNC117 and 10-1074 were measured using a bNAb-specific pseudovirus neutralization assay^18^. The average half-lives of 3BNC117 and 10-1074 were 14.9 and 20.3 days, respectively (*P* = 0.001; Fig. 1b, Extended Data Fig. 3a, Supplementary Tables 4, 5). There was no significant difference in antibody half-lives in the absence (group 1) or presence (group 2) of ART (Extended Data Fig. 3b, c), and the half-lives were similar to those obtained in previous clinical trials after one dose of each antibody during suppressive ART or three doses of each antibody during ART interruption (Extended Data Fig. 3d and refs. ^11–13^). We conclude that the pharmacokinetic profiles of 3BNC117 and 10-1074 are independent of concurrent antiretroviral medication during viral suppression and are not altered after multiple administrations over a period of 20 weeks.

## Viral suppression

**Fig. 2:**
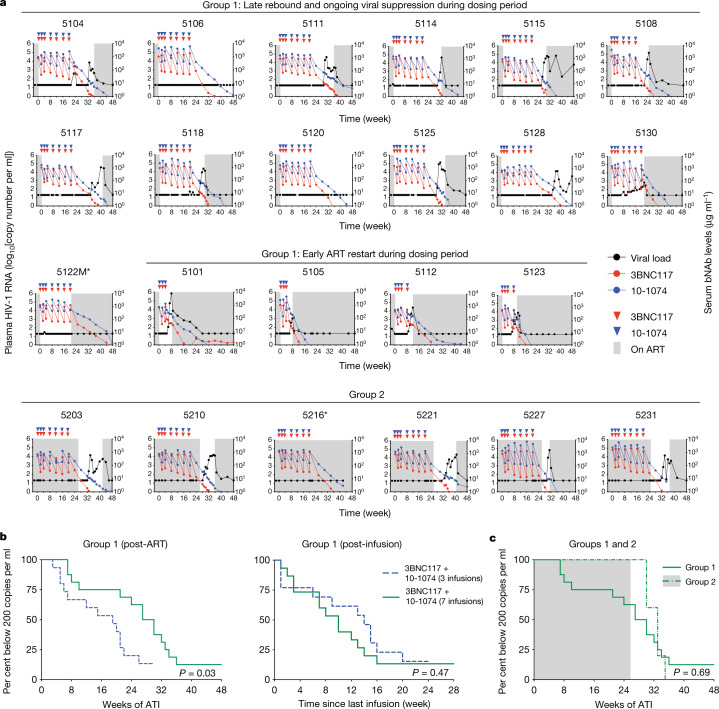
Virological and pharmacokinetic follow-up of individual participants. **a**, Plasma HIV-1 RNA levels (black line; left *y*-axis) and bNAb serum concentrations (3BNC117, red; 10-1074, blue; right *y*-axis) for group 1 participants who maintained viral suppression (*n* = 13) or resumed ART (*n* = 4) during the dosing period as well as group 2 (*n* = 6) participants. Red and blue triangles indicate 3BNC117 and 10-1074 infusions, respectively. Grey shaded areas indicate time on ART. The lower limit of detection of HIV-1 RNA was 20 copies per ml. Asterisk indicates participants with protocol deviations regarding analytical treatment interruption. Participant 5115 resumed ART before week 31 but did not achieve full viral suppression owing to non-compliance to a daily regimen. Participant 5128 resumed ART at week 48 and achieved full viral suppression at an additional week 52 study visit. **b**, Kaplan–Meier plots summarizing time to viral rebound after ART discontinuation (left) or after the last antibody infusions (right) for all group 1 participants receiving up to seven infusions (*n* = 16, green line). The dotted blue line indicates a cohort of individuals who received three infusions of the antibody combination^12^ (*n* = 15 participants). The *y*-axis indicates the percentage of participants who maintained viral suppression. The *x*-axis indicates the number of weeks after the start of analytical treatment interruption (ATI) (left) and the number of weeks after the last infusion (right). Participant 5122M was excluded from the analysis owing to COVID-19 related re-initiation of ART. Participant 5104 showed two viral blips of <500 copies per ml that were subsequently controlled before rebounding with sustained viraemia at week 33. **c**, Kaplan–Meier plots summarizing time to viral rebound for group 1 (green line) and group 2 (dotted green line) participants, respectively. Grey shaded area indicates time on ART for group 2 participants. Participant 5216 decided against ART interruption and was excluded from the analysis. Log–rank (Mantel–Cox) test was used to determine statistical significance in **b**, **c**.

Thirteen out of seventeen (76%) evaluable group 1 participants maintained viral suppression for at least 20 weeks after ART discontinuation. For the 16 group 1 participants who did not re-initiate ART before rebound, repeated combination antibody therapy was associated with viral suppression for a minimum of 7 weeks with a median time to rebound of 28.5 weeks (Fig. 2a, Supplementary Tables 4, 5). The individuals who received all seven bNAb infusions maintained viral suppression for 21 to more than 48 weeks with a median time to rebound of 32 weeks or 12 weeks after the last bNAb infusion. Although the overall time to rebound was significantly longer than in individuals who received only three infusions of the same antibodies^12^ (Fig. 2b, left, log-rank Mantel–Cox *P* = 0.03), the time to rebound after the last infusion was similar (Fig. 2b, right, log-rank Mantel–Cox *P* = 0.47). Two of the group 1 participants (5106 and 5120) completed study follow-up at 48 weeks without experiencing rebound. Participant 5106 was lost to follow-up after 1 year, and participant 5120 remains virally suppressed after 2 years in the absence of detectable antiretroviral drugs. Notably, participant 5106 is heterozygous for human leukocyte antigen (HLA) B57, which is associated with enhanced HIV control^19^.

The participants in group 2 maintained viral suppression for a median of 7 weeks (4–9 weeks) after ART was discontinued 13 weeks (range 10–15 weeks) after the last bNAb infusion (Fig. 2c). There was no statistically significant difference in time to viral rebound after ART interruption between group 1 and group 2 participants (Fig. 2c, log-rank Mantel–Cox *P* = 0.69).

The average serum concentration of 3BNC117 and 10-1074 at the time of rebound in individuals who remained suppressed after week 20 was 3.5 and 28.3 μg ml^−1^, respectively. Consistent with a previous report^12^, these participants experienced viral rebound only after the serum concentration of 3BNC117 decreased below approximately 10 μg ml^−1^, resulting in a period of 10-1074 monotherapy. We conclude that repeated infusions of two anti-HIV-1 bNAbs can maintain viral suppression for longer periods of time than previously reported as long as bNAb levels remain therapeutic. Similar results were observed in a parallel placebo-controlled clinical trial—sponsored by the National Institute of Allergy and Infectious Diseases of the National Institutes of Health (NIAID/NIH)—in which people living with HIV who started ART during the early phase of infection received eight doses of the same antibody combination over a 24-week period in the absence of ART^20^.

## Baseline and rebound antibody sensitivity

**Fig. 3:**
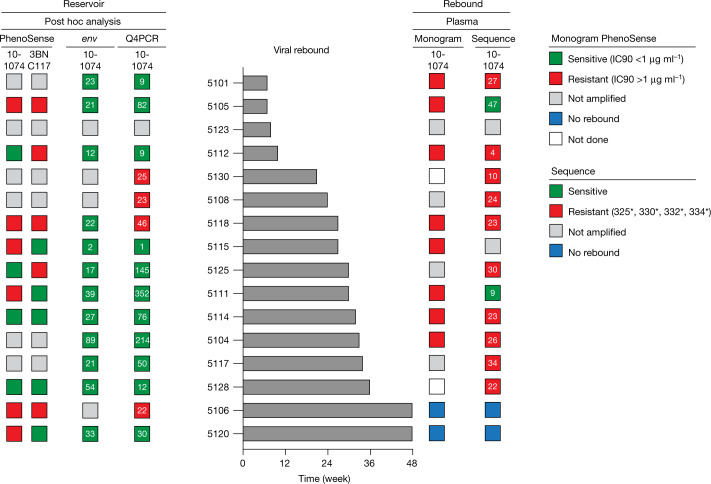
Post hoc sensitivity analysis of reservoir and plasma rebound viruses and impact on time to viral rebound. Latent reservoir (left) and plasma rebound viruses (right) from participants in group 1 (*n* = 16) were phenotypically and genotypically analysed for resistance to 10-1074 and 3BNC117, respectively (Methods). Sequence analysis was based on *env* sequences recovered by limiting-dilution PCR at baseline or by Q4PCR from all time points tested. The middle panel depicts time to viral rebound in ascending order. PhenoSense Monoclonal Antibody Assay antibody-sensitive (IC_90_ < 1 μg ml^−1^) and -resistant viruses (IC_90_ > 1 μg ml^−1^) are depicted with green and red squares, respectively. Sequence analysis of antibody-sensitive (absence of resistant viruses) or -resistant (identification of one or more resistant viruses) viruses are depicted in green and red squares, respectively. The number depicted in the squares represents the number of sequences analysed. Participant samples for which no results or sequences could be obtained are depicted in grey. Participants who maintained viral suppression without experiencing rebound are shown with a blue square. Samples that were not analysed are indicated with a white square.

Study participants were enrolled without prior screening for proviral reservoir sensitivity to 3BNC117 or 10-1074. To examine the potential effect of viral monoclonal antibody sensitivity testing on time to viral rebound, we performed post hoc analyses of reservoir and plasma rebound viruses. Envelope (*env*) sequences were obtained from plasma at the time of viral rebound or from the reservoir by limiting-dilution PCR and Q4PCR^21^ (see Methods). We analysed *env* sequences for known 10-1074-resistance mutations (Methods) and categorized them as sensitive or resistant. Sequence-based predictions of resistance were not possible for 3BNC117, and outgrowth assays are also limited in this respect owing to limited numbers of viruses and disparity between outgrowth and rebound viruses^12,22,23^. The samples were also analysed for resistance to 10-1074 and 3BNC117 by the PhenoSense Monoclonal Antibody Assay (Labcorp-Monogram Biosciences) with an exploratory susceptibility threshold (Fig. 3, Supplementary Table 6).

**Extended Data Fig. 4:**
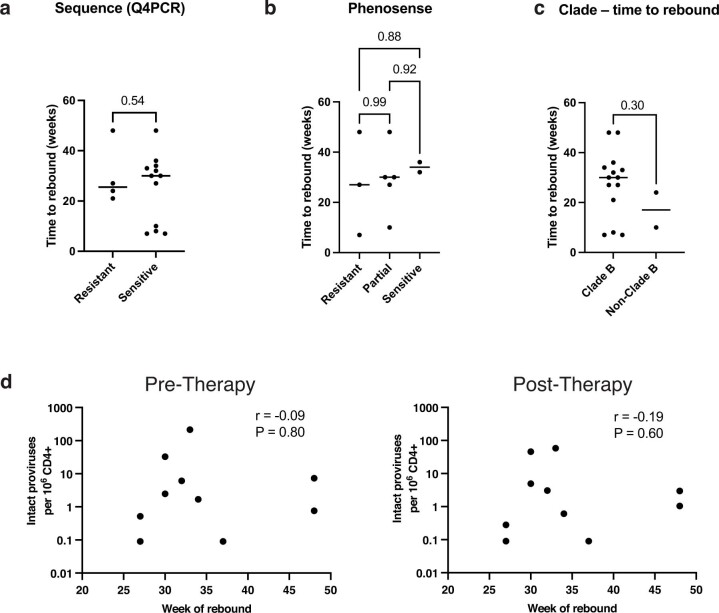
Time to viral rebound. **a**, Comparison of time to viral rebound between Group 1 participants with genotypically 10-1074 resistant or sensitive reservoir viruses recovered by Q4PCR. Black line depicts median. Statistical significance was determined using a two-tailed unpaired t-test. **b**, Comparison of time to viral rebound between Group 1 participants with phenotypically resistant (n = 3), partially resistant (n = 5) or sensitive (n = 2) reservoir viruses to 3BNC117 and 10–1074 by PhenoSense. Black line depicts median. Statistical significance was determined using one-way ANOVA with Tukey multiple comparison test. **c**, Comparison of time to viral rebound between Group 1 that harbor clade B (n = 15) and non-clade B viruses (n = 2), respectively. Black line depicts median. Statistical significance was determined using a two-tailed unpaired t-test. **d**, Intact proviral frequency per 10^6^ CD4 T cells versus time of viral rebound in the Group 1 participants that remained suppressed after week 20 (n = 10). Graphs show the correlation at pre- (left) and post-therapy (right) time points, respectively. The r and P values were determined by two-tailed Pearson’s correlations.

Sequence analysis of 16 group 1 participants for 10-1074-resistance mutations was performed by limiting-dilution *env* PCR (*n* = 360) and by Q4PCR (*n* = 1,096). Although 10-1074-resistance mutations were not detected in the more limited *env* PCR sample, Q4PCR detected four individuals (25%) with 10-1074-resistant latent viruses (Fig. 3, Supplementary Table 7). The time to rebound after ART interruption in individuals harbouring 10-1074-resistant proviruses was shorter but not statistically different from their sensitive counterparts (*P* = 0.54, 25.5 versus 30 weeks; Extended Data Fig. 4a). By contrast, limiting-dilution *env* PCR on plasma rebound viruses that emerged after a period of 10-1074 monotherapy showed 10-1074 target site mutations in all but two group 1 participants who rebounded (Fig. 3, Supplementary Table 7).

The PhenoSense assay did not produce results for reservoir viruses in 38% of the group 1 participants (Fig. 3, Supplementary Table 6). Results were available for ten group 1 participants. Median time to rebound was not statistically different in participants with sensitive, single- or double-resistant reservoir viruses (one-way analysis of variance (ANOVA) *P* = 0.89, Extended Data Fig. 4b). If individuals whose samples did not produce results in this assay and those with resistant reservoir viruses were excluded from participation, then 14 out of the 16 group 1 participants would have been excluded (Figs. 2a, 3, Supplementary Table 6). Thus, in this small dataset, both genotypic and phenotypic sensitivity analyses could not predict clinical outcome and time to viral rebound. Amplification failure, sequencing depth, disparity between outgrowth and rebound viruses, poorly defined sensitivity cut-off values and our inability to reliably define the sequences that result in 3BNC117 resistance limit the applicability of currently available reservoir screening assays. Additional analysis of the utility of these assays will require larger datasets and analysis of prospective as well as retrospective studies.

## Latent reservoir

**Fig. 4:**
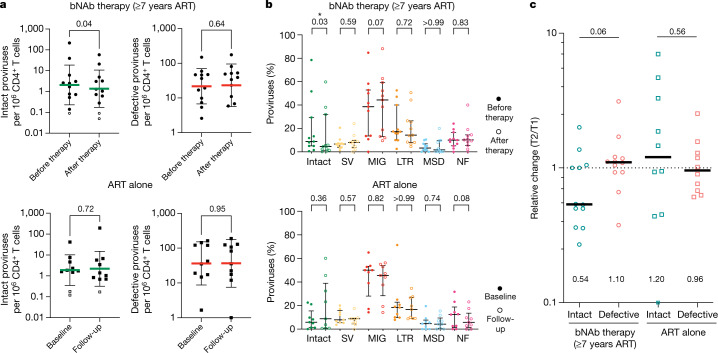
Reservoir quantification and composition. **a**, Frequency of intact and defective proviral genomes per 10^6^ CD4^+^ T cells (log normalized) as determined by Q4PCR pre-therapy and post-therapy (26 weeks) for bNAb therapy with participants who have been on ART for at least 7 years (top, circles) and baseline and follow-up (24–58 weeks) for ART-alone (bottom, squares) groups, respectively. All participants with paired reservoirs measurements were included (bNAb therapy with at least 7 years ART *n* = 12, ART alone *n* = 10). Open symbols represent lower limit of detection (defined as half of intact proviral frequency assuming one intact proviral genome in the total number of analysed cells without target identification). Green and red horizontal bars depict mean ± s.d. of intact and defective proviral frequencies, respectively. **b**, Longitudinal changes in relative representation of proviral subtypes in the bNAb therapy with at least 7 years ART (top) and ART-alone (bottom) groups, respectively. Depicted are the fractions of intact and defective proviral subtypes relative to total proviruses recovered from each participant at the indicated time point. Proviral subtypes: intact; SV, structural variation; MIG, missing internal gene; LTR, LTR defects; MSD, major splice donor mutation; and NF, non-functional. Participants in whom at least one intact proviral genome was recovered are shown (bNAb therapy with at least 7 years ART *n* = 11, ART alone *n* = 9). Horizontal bars show median and interquartile range. **c**, Relative change in proviral frequencies (indicated at the bottom of each dataset) between pre-therapy (T1) and post-therapy time points (T2) for intact (green) and defective (red) proviral frequencies in bNAb therapy (circles) and ART-alone (squares) groups, respectively. Horizontal bars indicate median change. Open square with cross represents individual data point outside the *y*-axis range (relative change TSC − 127 = 0.09). *P*-values are shown at the top of graphs and were determined using two-tailed paired Student’s *t*-test in **a** and two-tailed Wilcoxon matched-pairs signed-rank test in **b**, **c**.

To examine the effect of prolonged bNAb therapy on the latent reservoir, we performed Q4PCR on samples obtained approximately 2–4 weeks before and 24 or 26 weeks after the first antibody infusion^21,24^ (bNAb therapy group, Supplementary Tables 1 and 8). In addition, we assayed the reservoir in the parallel group of 10 participants that remained on suppressive ART without receiving antibody infusions at paired time points with a median interval of 32 weeks (range 24–58 weeks) between baseline and follow-up (ART-alone group; Fig. 4, Supplementary Tables 2, 8).

**Extended Data Fig. 5:**
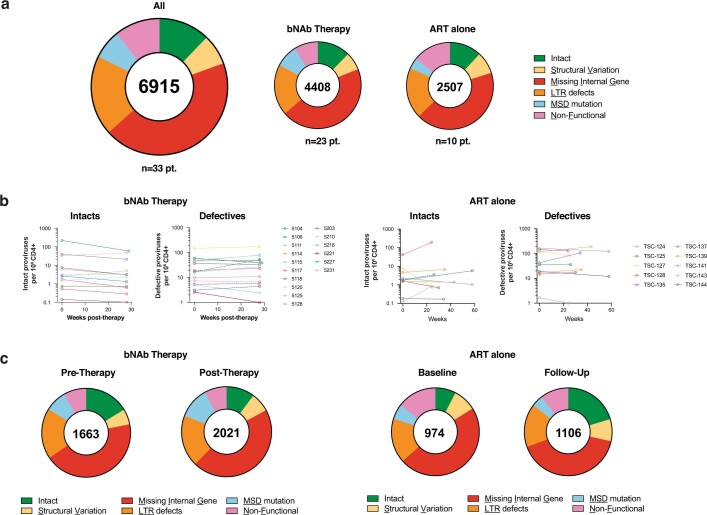
Reservoir sequence analysis. **a**, Pie charts depict the distribution of recovered intact and defective proviral sequences. Left pie chart shows the distribution of all 6915 recovered from 33 participants. Middle and right pie charts show the distribution for participants in the bNAb Therapy and ART alone groups, respectively. The number in the middle of the pie represents the total number of proviruses sequenced. Pie slices indicate the proportion of sequences that were intact or had different defects, including structural variation (yellow), missing internal genes (red), LTR defects (orange), MSD mutation (blue) and non-functional (pink). **b**, Graph depicts the paired measurements of the intact and defective proviral reservoir size in the bNAb Therapy (left panel) and ART alone (right panel) groups. Y-axis shows frequencies of intact or defective proviruses per 10^6^ CD4+ T cells. X-axis depicts time in weeks. Paired measurements with a relative decrease or increase are shown with a solid or dotted line, respectively. **c**, Pie charts depict the distribution of recovered intact and defective proviral sequences at pre- and post-therapy (bNAb Therapy group, left panel) or baseline and follow-up time point (ART alone group, right panel). The number in the middle of the pie represents the total number of proviruses sequenced. Only participants for whom at least one intact proviral genome was recovered are included (bNAb Therapy n = 14, ART alone n = 9).

**Extended Data Fig. 6:**
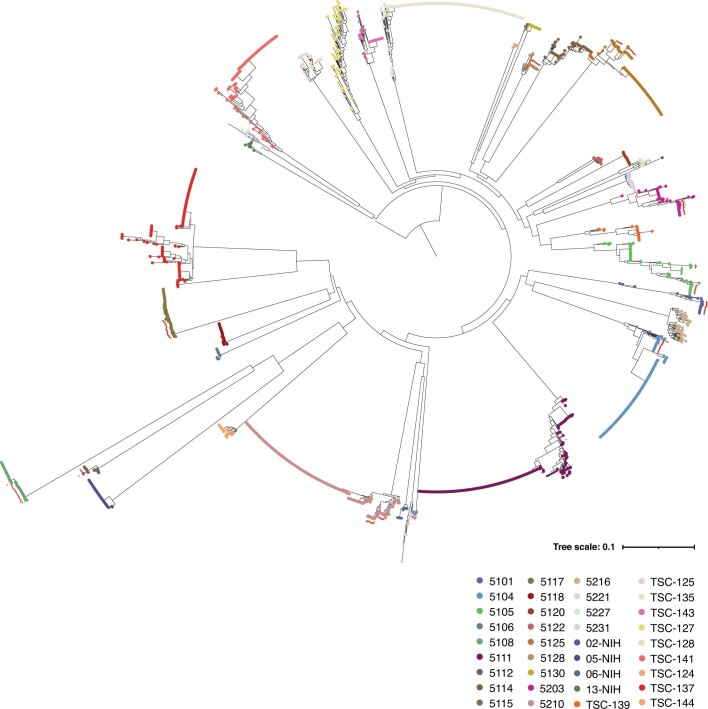
Phylogenetic tree of reservoir and rebound viruses. Maximum likelihood phylogenetic trees of complete env sequences obtained from 36 participants (including sequences recovered from NIH/NIAID participants; no complete env sequence obtained from participant 5123) by Q4PCR and when available rebound viruses from SGA. Participants are indicated in individual colours. Red triangles indicate rebound viruses.

Overall, we recovered and analysed 6,074 defective (bNAb therapy *n* = 3,866, ART alone *n* = 2,208) and 841 intact proviral (bNAb therapy *n* = 542, ART alone *n* = 299) sequences (Extended Data Fig. 5a, Supplementary Table 8). Five different categories of defective proviruses were documented: (1) structural variation; (2) missing internal genes; (3) long terminal repeat (LTR) defects; (4) major splice donor mutation; and (5) non-functional (see Methods). In agreement with previous work, the most abundant categories of defective proviruses were those with missing internal genes due to internal deletions of varying size (Extended Data Fig. 5a and refs. ^21,25^). Phylogenetic analysis showed that the trial participants were infected with epidemiologically distinct viruses (Extended Data Fig. 6). Two participants (5108 and 5112) were infected with non-clade B subtypes G and A1, respectively. The median time to viral rebound was shorter for participants harbouring non-clade B viruses but the difference did not reach statistical significance (*P* = 0.30, 17 weeks versus 30 weeks; Extended Data Fig. 4c). Moreover, time to rebound was not directly correlated with the size of the intact proviral reservoir (Extended Data Fig. 4d).

Reservoir half-life appears to increase after seven years of ART therapy^26^, and individuals in our cohort who received bNAb therapy had a shorter median time on uninterrupted ART than those that did not receive antibody treatment (Extended Data Fig. 1c). To control for this potentially confounding variable, we omitted all individuals who had been on suppressive ART for less than seven years from our initial reservoir analysis.

As expected, there was no overall change in the absolute number of defective proviruses in the reservoir during the observation period in either the active therapy or the parallel ART-alone group^25–28^ (Fig. 4a–c, Extended Data Fig. 5b, c, Supplementary Table 8). In addition, there was also no significant change in the relative representation of the five different categories of defective proviruses in the active therapy or parallel ART-alone group (Fig. 4b, Extended Data Fig. 5b, c, Supplementary Table 8).

In contrast to the defective proviruses, there was a moderate but significant change in both the absolute number and relative representation of intact proviruses in the antibody-treated cohort (*P* = 0.04 and *P* = 0.03 respectively, bNAb therapy with at least 7 years ART; Fig. 4a, b). For the active therapy group, there was a median 46% reduction in the intact reservoir over 6 months that was close to but did not reach statistical significance (*P* = 0.06; Fig. 4c). Additional time points would be required to calculate the half-life of the reservoir in individuals who received immunotherapy.

**Extended Data Fig. 7:**
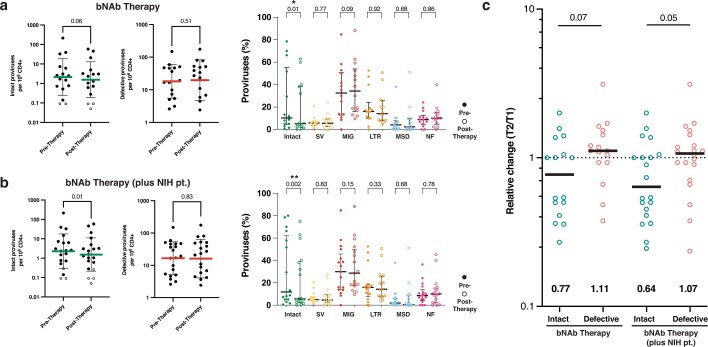
Reservoir quantification and composition subgroup analyses. **a–c**, Subgroup reservoir analyses for bNAb Therapy group (n = 16) (**a**); bNAb Therapy group (plus NIH pt., n = 20) including 4 additional participants from a corresponding NIH/NIAID clinical trial (**b)**. Left panels show the frequency of intact and defective proviral genomes per 10^6^ CD4+ T cells (log normalized) as determined by Q4PCR pre-therapy and post-therapy (24–26 weeks) for each respective subgroup. Open symbols represent lower limit of detection (defined as half of intact proviral frequency assuming 1 intact proviral genome/total number of analysed cells without target identification). Green and red horizontal bars depict the mean with SD of intact and defective proviral frequencies, respectively. Statistical significance was determined using two-tailed paired Student’s t-test. Right panels show longitudinal changes in relative representation of proviral subtypes in the respective bNAb Therapy subgroups. Depicted are the fractions of intact and defective proviral subtypes relative to total proviruses recovered from each participant at the indicated time point. Proviral subtypes: Intact, SV, Structural Variation; MIG, Missing Internal Gene; LTR, LTR defects; MSD, Major Splice Donor mutation; NF, Non-Functional. Only participants for whom at least one intact proviral genome was recovered are shown (bNAb Therapy group n = 14; bNAb Therapy group plus NIH pt. n = 17). Horizontal bars show median and interquartile range (IQR). Statistical significance was determined using two-tailed Wilcoxon matched-pairs signed rank test. **c**, Relative change in proviral frequencies between pre-therapy (T1) and post-therapy time points (T2) for intact (green) and defective (red) proviral frequencies in bNAb Therapy subgroups. Plotted values depict relative change with the black horizontal bars showing the median change. Statistical significance was determined using two-tailed Wilcoxon matched-pairs signed-rank test for matched comparisons within bNAb Therapy subgroups, respectively.

To further pressure-test the results we performed two additional analyses: (1) all participants irrespective of time on ART; and (2) all participants plus all volunteers (*n* = 4) from a parallel NIH/NIAID-sponsored clinical trial that had a measurable reservoir and maintained viral suppression in the absence of ART after receiving eight doses of the same antibody combination over a 24-week period^53^ (Extended Data Fig. 7a–c, Supplementary Tables 1, 8). In no instance was there a significant change in the defective reservoir. By contrast, when all of the individuals in our cohort were included, there was a decrease in the relative percentage (*P* = 0.01), absolute numbers (*P* = 0.06) and relative numbers (*P* = 0.07) of intact proviruses (Extended Data Fig. 7a, c). When the additional volunteers from NIH/NIAID were also included, relative percentage (*P* = 0.002), absolute numbers (*P* = 0.01) and relative numbers (*P* = 0.05) of intact proviruses also decreased (Extended Data Fig. 7b, c).

Notably, the relative representation of non-functional and major splice donor mutation defective proviruses—near-full length HIV genomes that differ from intact proviral genomes by as little as one nucleotide substitution—does not change significantly after antibody therapy in any of the groups analysed. Therefore, long-distance PCR amplification efficiency cannot account for the observed changes in the intact proviral reservoir of the bNAb therapy group (Fig. 4b, Extended Data Fig. 7a, b).

**Extended Data Fig. 8:**
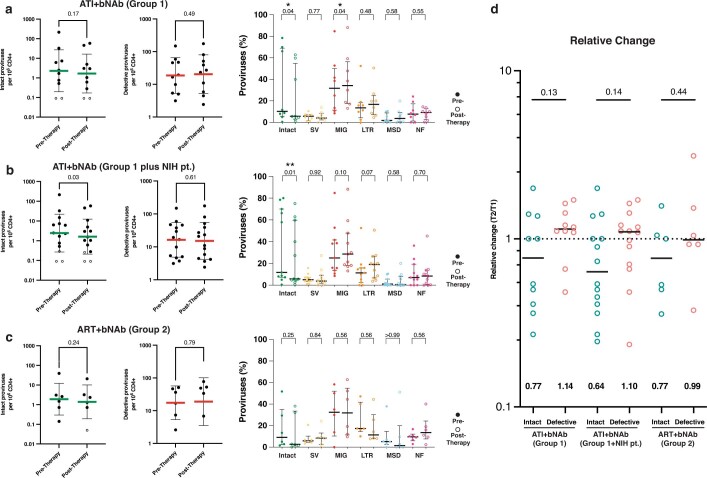
Reservoir quantification and composition ATI+bNAb versus ART+bNAb. **a–c**, Subgroup reservoir analysis for Group 1 participants that interrupted ART (ATI+bNAb n = 10) (**a**), for Group 1 and 4 additional participants from a corresponding NIH/NIAID clinical trial (ATI+bNAb Group 1 plus NIH pt., n = 14) (**b**), and Group 2 participants that remained on ART (ART+bNAb, n = 6) during bNab therapy (**c**). Left panels show the frequency of intact and defective proviral genomes per 10^6^ CD4+ T cells (log normalized) as determined by Q4PCR pre-therapy and post-therapy (24–26 weeks). Open symbols represent lower limit of detection (defined as half of intact proviral frequency assuming 1 intact proviral genome/total number of analysed cells without target identification). Scatter plots depict mean with SD. Statistical significance was determined using two-tailed paired Student’s t-test. Right panels show longitudinal changes in relative representation of proviral subtypes in the respective subgroups. Depicted are the fractions of intact and defective proviral subtypes relative to total proviruses recovered from each participant at the indicated time point. Proviral subtypes: Intact, SV, Structural Variation; MIG, Missing Internal Gene; LTR, LTR defects; MSD, Major Splice Donor mutation; NF, Non-Functional. Only participants for whom at least one intact proviral genome was recovered are shown (ATI+bNAb Group 1 n = 8; ATI+bNAb Group 1 plus NIH pt. n = 11, ART+bNAb Group 2 n = 6). Horizontal bars show median and interquartile range (IQR). Statistical significance was determined using two-tailed Wilcoxon matched-pairs signed rank test. **d**, Relative change in proviral frequencies between pre-therapy (T1) and post-therapy time points (T2) for intact (green) and defective (red) proviral frequencies for ATI+bNab Group 1 (left two columns), ATI+bNAb Group 1 plus NIH (middle two columns), and ART+bNab (right two columns) participants, respectively. Plotted values depict relative change with the black horizontal bars showing the median change. Statistical significance was determined using two-tailed Wilcoxon matched-pairs signed-rank test for matched comparisons within groups.

To determine whether the observed changes in the intact proviral reservoir are dependent on analytical treatment interruption, we performed a separate analysis of specimens from participants in whom treatment was interrupted during bNAb dosing (Extended Data Fig. 8a, b, d) and group 2 participants who remained on ART (Extended Data Fig. 8c, d).

Subgroup analysis of participants who underwent analytical treatment interruption (ATI) (ATI plus bNAb group 1 and ATI plus bNAb group 1 plus participants from the parallel NIH/NIAID-sponsored clinical trial  (NIH pt.); Extended Data Fig. 8a, d and Extended Data Fig. 8b, d, respectively) showed the same direction and magnitude of absolute and relative change in the proviral landscape as the overall analysis (ATI plus bNAb group 1, absolute number *P* = 0.17, relative composition *P* = 0.04, relative change *P* = 0.13, median reduction 23%; ATI plus bNAb group 1 plus NIH pt., absolute number *P* = 0.03, relative composition *P* = 0.01, relative change *P* = 0.14, median reduction 36%; Extended Data Fig. 8a, b, d).

Similar changes in the reservoir were observed in participants who remained on ART during antibody infusions (ART plus bNAb group 2), but the limited sample size did not reach statistical significance (*n* = 6, absolute number *P* = 0.24, relative composition *P* = 0.25, relative change *P* = 0.44, median reduction 23%, Extended Data Fig. 8c, d). We conclude that the size and composition of the intact latent reservoir changes during a six-month interval in individuals who maintain suppression while receiving repeated doses of anti-HIV-1 bNAbs.

Sequence-based reservoir analyses allow for the unambiguous identification of intact proviruses but, owing to the relative scarcity of intact proviruses in the latent reservoir, the analysis inevitably relies on small numbers and will need additional confirmation.

The results of this open-label study in chronically infected participants and the parallel placebo-controlled clinical trial in individuals who initiated ART during primary infection^53^ ﻿demonstrate that antibodies can maintain HIV-1 suppression for prolonged periods of time. Combinations of antibodies targeting non-overlapping epitopes appear to be essential in this respect because of the emergence of antibody resistance during monotherapy^11,13^. In 76% (13 out of 17) of the limited number of individuals examined, most of whom were infected with clade B HIV-1, the 3BNC117 plus 10-1074 antibody combination maintained suppression in the absence of pre-screening for sensitivity. A key challenge in the further implementation of antibody therapy is that available combinations do not cover 100% of all HIV-1 strains. In addition, antibody sensitivity testing remains suboptimal, in part because the depth of the reservoir makes it difficult to obtain fully representative samples. Although these are not yet available, careful selection of dosing regimens to avoid periods of antibody monotherapy, close monitoring and education of participants with regards to risks associated with viral rebound during ART interruption studies are of utmost importance^29^. Despite these current challenges, long-acting antibodies may become a viable therapeutic option in combination with long-acting ART to achieve higher rates of sustained viral suppression among sub-populations of people living with HIV who face challenges with daily regimens.

The HIV-1 reservoir is composed of very large numbers of defective and intact proviruses integrated into a variety of genome locations^30–32^. Reactivation of intact proviruses in ART-suppressed individuals in whom treatment has been interrupted leads to rebound viraemia after two to four weeks in most individuals, even after periods of prolonged ART^4^. It has been estimated that a three to four orders of magnitude decrease in reservoir size would be required to alter rebound kinetics^33^. bNAb therapy appears to accelerate intact reservoir decay but, as expected, the magnitude of the change in reservoir size after six months was not sufficient to delay rebound.

Several methods focusing on intact proviruses have been used to characterize the reservoir, including quantitative^34^ and qualitative^35^ viral outgrowth assays, near full-length PCR^36,37^, intact proviral PCR^27,38^ (IPDA), and Q4PCR^21^. Although the absolute number of intact proviruses detected by each of these methods varies^39^, they all report the same intact proviral reservoir half-life of between four and five years^25–27,40,41^. Defective proviruses, which are one to two orders of magnitude more abundant than intact proviruses, have a much longer half-life than intact proviruses^26,28,37,42^. The discrepancy between the size and half-life of the intact and defective reservoirs makes it essential that interventions aimed at altering the intact reservoir are measured by methods that can definitively distinguish between these two compartments.

Sequencing studies revealed that the intact reservoir is largely composed of expanded clones of CD4^+^ T cells. Individual clones are dynamic and as the magnitude of the intact reservoir decays, it becomes more clonal and less complex^25,31,35,40^. Similar to other T cells, latent provirus-containing CD4^+^ T cells undergo clonal expansion in response to antigen, including antigens associated with chronic viral infections such as cytomegalovirus^43,44^. Clonal expansion requires cellular activation and NF-κB expression, which also has the potential to reactivate latent proviruses^45^. Although high-level HIV-1 expression results in cell death^46^, in vitro experiments have shown that only a fraction of dividing latent cells die after reactivation^37,47,48^. Some of those that survive do so after transiently expressing the HIV-1 envelope protein or peptide–major histocompatibility complex complexes on the cell surface making them potential targets for bNAb^9,49,50^ or CD8^+^ T cell-mediated clearance^9,50^, as documented in pre-clinical studies in bNAb-treated SHIV-infected macaques^8,9^.

Our data suggest that anti-HIV-1 antibodies can maintain suppression and may accelerate the rate of reservoir decay. Antibody therapies might alter reservoir dynamics by limiting clonal expansion by targeting dividing cells that express viral proteins directly or by enhancing CD8^+^ T cell immunity. If these mechanisms are responsible for the antibody mediated changes in the reservoir, they might also occur under ART therapy. Larger studies of longer duration will be required to determine the precise half-life of the intact reservoir during antibody therapy and whether addition of antibodies to standard ART will affect reservoir half-life and contribute to strategies aimed at long term HIV-1 remission.

## Methods

### Study design

We conducted a phase 1b, open-label, randomized clinical trial of two treatment groups of chronically infected people living with HIV-1 (http://www.clinicaltrials. gov; NCT03526848). Study participants were enrolled sequentially according to eligibility criteria and randomized to either group 1 or group 2 in a 3:1 ratio. The planned sample size was 40 participants; however, owing to the COVID-19 pandemic, enrolment was stopped early with 26 participants, with 18 enrolled in group 1 and 8 in group 2. Participants received seven infusions of 3BNC117 and 10-1074 intravenously at a dose of 30 mg per kg body weight of each antibody, at weeks 0, 2 and 4, 8, 12, 16 and 20 unless viral rebound occurred. Viral rebound was defined as plasma HIV-1 RNA level > 200 copies per ml in two consecutive measurements. Group 1 discontinued ART 2 days after the first antibody infusions, whereas group 2 remained on ART before discontinuing the medication 26 weeks after the first antibody infusions. During the treatment interruption periods of the study, ART was resumed if there was a confirmed >30% decline in CD4^+^ T cell count or confirmed CD4^+^ T cell count decrease to < 350 cells per μl from baseline (day 0). Plasma HIV-1 viral RNA levels were monitored weekly through study week 38 and virologic criteria for ART re-initiation accommodated different phases of the follow-up period: (part A) week 0 to week 26, 2 consecutive plasma HIV-1 RNA levels > 200 copies per ml measured weekly, (part B) week 26 to week 38, sustained (>4 weeks) HIV-1 RNA levels > 1,000 copies per ml measured weekly; and (part C) week 38 to week 48, 2 consecutive plasma HIV-1 RNA levels > 1,000 copies per ml measured every other week. Other ART restart criteria included pregnancy and symptoms consistent with acute retroviral syndrome. Study participants were followed for 48 weeks after the first infusions. The study hypotheses were that the administration of repeated infusions of 3BNC117 and 10-1074 in the absence of ART would be safe and well tolerated, maintain viral suppression in people living with HIV during ATI and interfere with the HIV-1 reservoir. The primary endpoints of the study were: (1) rate of viral rebound 12 weeks after ART interruption (week 12 for group 1, and week 38 for group 2); (2) time to return of viraemia (first plasma HIV-1 RNA level > 200 copies per ml of 2 consecutive measurements) after ART interruption; (3) size of the proviral HIV-1 reservoir before and after 7 antibody infusions; and (4) rate and severity of adverse events and serious adverse events. All participants provided written informed consent before participation in the study and the study was conducted in accordance with good clinical practice. The protocol was approved by the US Food and Drug Administration and the Rockefeller University and Mass General Brigham Human Research Institutional Review Boards (IRBs).

### Study participants

Study participants were recruited at the Rockefeller University Hospital, New York, USA, and the Massachusetts General Hospital, Boston, USA. Eligible participants were adults aged 18–65 years, HIV-1-infected, on ART with plasma HIV-1 RNA levels of <50 copies per ml for at least 12 months (one viral blip of >50 but <500 copies per ml during this period was allowed), plasma HIV-1 RNA levels < 20 copies per ml at screening visit, reported or confirmed CD4^+^ T cell count nadir of > 200 cells per μl and a current CD4^+^ T cell counts > 500 cells per μl. Participants on an ART regimen that included a NNRTI were switched to an integrase inhibitor-based regimen (dolutegravir plus tenofovir disoproxil fumarate and emtricitabine) at least four weeks before treatment interruption due to the prolonged half-life of NNRTIs. Exclusion criteria included medical history of resistance to two or more classes of antiretroviral medication, prior anti-HIV-1 monoclonal antibody therapy, concomitant hepatitis B or C infection, clinically relevant physical findings, medical conditions or laboratory abnormalities and pregnancy or lactation. Time to viral rebound was defined as the first of two consecutive viral loads of >200 copies per ml with the exception of participant 5104 who experienced two viral blips (two viral loads of <500 copies per ml) which were subsequently followed by suppressed viraemia before rebounding with sustained viraemia at week 33.

As a result of the COVID-19 pandemic, one participant in group 1 (5122M) re-initiated ART despite not having experienced viral rebound and one participant in group 2 (5216) decided against analytical treatment interruption after completion of all seven antibody infusions. Participant 5126M was withdrawn after the first antibody infusion because his NNRTI-based ART regimen was inadvertently not switched according to protocol and he was found to have a viral load of 615 copies per ml at baseline (day 0). In addition, two participants (5207 and 5219) opted to withdraw from the study for COVID-19-related and other personal reasons.

Peripheral blood mononuclear cell (PBMC) samples from four out of seven study participants in a parallel NIH/NIAID-sponsored clinical trial who received 3BNC117 in combination with 10-1074 during ART interruption (http://www.clinicaltrials.gov; NCT03571204) were included for reservoir assessments (Sneller et al., submitted manuscript). This trial included participants that initiated ART within 90 days of being diagnosed with acute or early HIV infection. Enrolled participants discontinued ART 3 days after the first antibody infusions and received a total of 8 3BNC117 and 10-1074 infusions over 24 weeks. Reservoir assessments were performed at baseline and week 24. The 4 participants were selected for analysis because they had measurable reservoirs and maintained viral suppression while receiving all 8 infusions of 3BNC117 and 10-1074 (30 mg per kg body weight of each antibody) over a period of 24 weeks. One additional participant who maintained viral suppression over 24 weeks did not have a measurable reservoir by Q4PCR and was excluded from the analysis. The protocol was approved by the Institutional Review Board of the National Institute of Allergy and Infectious Diseases, National Institutes of Health (Sneller et al., submitted manuscript).

Ten additional individuals on suppressive ART and with similar baseline clinical characteristics were followed over time under a parallel observational protocol for repeated blood donations and reservoir assessments while remaining on suppressive ART (ART-alone group; Extended Data Fig. 1). Clinical data collection and management were carried out using the software iRIS by iMedRIS Version 11.02.

### Study procedures

3BNC117 and 10-1074 were prepared according to the Rockefeller University Pharmacy and MGH Pharmacy Standard Operating Procedures and administered intravenously at a dose of 30 mg kg^−1^. Monoclonal antibody intravenous infusions were administered sequentially, each antibody over 60 min. Study participants were observed at the Rockefeller University Hospital or the Massachusetts General Hospital for 1 h after the last antibody infusion.

Participants returned for weekly (part A and part B) and bi-weekly (part C) follow-up visits during the ATI period for safety assessments, which included physical examination and measurements of clinical laboratory parameters such as haematology, chemistries, urinalysis and pregnancy tests (for women). Viral load was monitored on a weekly (weeks 1 to 38) or bi-weekly (weeks 38–48) basis and CD4^+^ T cell counts were monitored every 1–2 weeks during analytical treatment interruption and 2-4 weeks after ART re-initiation. During every study visit, participants were counselled of the importance to use barrier protection while off ART and until viral load became undetectable after ART re-start to decrease the risk of HIV transmission to a sexual partner. Two participants acquired HCV during study follow-up (one probable sexual transmission after ART had been resumed but prior to viral suppression and one linked to drug use during ATI) (Supplementary Table 3). The protocol was modified to include monthly testing for bacterial sexually transmitted infections while participants remained off ART and until viral suppression after ART restart. Study investigators evaluated and graded adverse events according to the Division of AIDS (DAIDS) Table for Grading the Severity of Adult and Pediatric Adverse Events version 2.1 (July 2017) and determined causality.

Leukapheresis was performed at the Rockefeller University Hospital or at the Massachusetts General Hospital at week −2 and week 26. Blood samples were collected before and at multiple times after 3BNC117 and 10-1074 infusions. Samples were processed within 4 h of collection, and serum and plasma samples were stored at −80 °C. PBMCs were isolated by density gradient centrifugation. The absolute number of PBMCs was determined using an automated cell counter (Vi-Cell XR; Beckman Coulter) or manually, and cells were cryopreserved in fetal bovine serum plus 10% DMSO before storage in liquid nitrogen.

### Plasma HIV-1 RNA levels

HIV-1 RNA levels in plasma were measured at the time of screening, at week −2, day 0 (before infusion), weekly during ATI, and every two weeks to every eight weeks after viral rebound had occurred. HIV-1 RNA levels were determined using the Roche COBAS AmpliPrep/COBAS TaqMan HIV-1 Assay (version 2.0) or the Roche cobas HIV-1 quantitative nucleic acid test (cobas 6800), which quantitate HIV-1 RNA over a range of 2 × 10^1^ to 1 × 10^7^ copies per ml. These assays were performed at LabCorp or at the local laboratory at Massachusetts General Hospital.

### CD4^+^ T cells

CD4^+^ T cell counts were determined by a clinical flow cytometry assay, performed at LabCorp or at the local laboratory at Massachusetts General Hospital.

### Measurement of 3BNC117 and 10-1074 serum levels

Serum concentrations of active 3BNC117 and 10-1074 were determined before and at the end of each 10-1074 infusion, every 4 weeks during the ATI period, every 4 to 8 weeks in the follow-up period after ART re-initiation, and at the time of viral rebound by a validated luciferase-based neutralization assay in TZM-bl cells as previously described^18^. In brief, serum samples were tested using a primary 1:20 dilution with a fivefold titration series against HIV-1 Env pseudoviruses Q842.d12 and X2088_c9, which are highly sensitive to neutralization by 3BNC117 and 10-1074, respectively, while fully resistant against the other administered antibody. In the case of the post-infusion time points of 10-1074, instances where the serum inhibitory dose (ID_50_) titres against X2088_c9 were >100,000, serum samples were also tested against a less sensitive strain, Du422. Similarly, in the case of the post-infusion time points of 3BNC117, instances where serum ID_50_ titres against Q842.d12 were >100,000, serum samples were also tested against a less sensitive strain, Q461.e2 (Supplementary Tables 4 and 5). To generate standard curves, 3BNC117 and 10-1074 clinical drug products were included in every assay set-up using a primary concentration of 10 μg ml^−1^ with a fivefold titration series. Serum concentrations of 3BNC117 and 10-1074 for each sample were calculated as follows: serum ID_50_ titre (dilution) × 3BNC117 IC_50_ or 10-1074 IC_50_ titre (μg ml^−1^) = serum concentration of 3BNC117 or 10-1074 (μg ml^−1^). Env pseudoviruses were produced using an ART-resistant backbone vector that reduces background inhibitory activity of antiretroviral drugs if present in the serum sample (SG3ΔEnv/K101P.Q148H.Y181C; M. S. Seaman, unpublished data). Virus pseudotyped with the envelope protein of murine leukemia virus (MLV) was used as a negative control. Antibody concentrations were calculated using the serum ID_80_ titre and monoclonal antibody IC_80_ if non-specific activity against MLV was detected. The lower limits of detection were 0.007 µg ml^−1^ and 0.005 µg ml^−1^ for 3BNC117 and 10-1074, respectively. All assays were performed in a laboratory meeting GCLP standards.

### Antiretroviral drug screening

Plasma samples were screened for a panel of antiretrovirals (tenofovir (TFV), emtricitabine (FTC), lamivudine (3TC), abacavir (ABC), zidovudine (ZDV), nevirapine (NVP), amprenavir (APV), atazanavir (ATV), darunavir (DRV), etravirine (ETR), elvitegravir (EVG), efavirenz (EFV), lopinavir (LPV), ritonavir (RTV), raltegravir (RTG), dolutegravir (DTG), maraviroc (MVC) and rilpivirine (RPV)) by the University of North Carolina Center for AIDS Research Clinical Pharmacology and Analytical Chemistry Core. Results were reported as ‘peak’ or ‘no peak’. No peak results correspond to a non-detectable antiretroviral concentration in the sample of >1 ng ml^−1^ for TFV, FTC, 3TC, ABC, ZDV and NVP, or >10 ng ml^−1^ for APV, ATV, DRV, ETR, EVG, EFV, LPV, RTV, RTG, DTG, MVC and RPV. Samples from participants who continued to maintain viral suppression for >12 weeks (group 1) or >12 weeks from last dose (group 2) were tested at approximately 12-week intervals until viral rebound. An additional sample from participant 5120 collected approximately 24 months after ART interruption was also tested.

### Phenotypic characterization and surface staining

Frozen PBMCs were thawed and washed with PBS. Cells were then surface-stained for 30 min in the dark at 4 °C with viability reagent (BD Horizon Fixable Viability Stain; BD Biosciences) and a 13-colour cocktail of monoclonal antibodies containing surface antibodies against CD19 (clone SJ25C1, dilution 1/250), CD20 (clone 2H7, dilution 1/100), CD66b (clone G10F5, dilution 1/250), CD14 (clone M5E2, dilution 1/150), CD3 (clone SK7, dilution 1/100), CD4 (clone OKT4, dilution 1/200), CD8 (clone RPA-T8, dilution 1/500), CD38 (clone HIT2, dilution 1/250), CD69 (clone FN50, dilution 1/500), CD71 (clone M-A712, dilution 1/100), CD25 (clone 2A3, dilution 1/300), CD152 (clone BNI3, dilution 1/250), CD279 (clone EH12.1, dilution 1/500), TIGIT (clone 741182, dilution 1/500), CD223 (clone T47-530, dilution 1/250) and CD336 (clone 7D3, dilution 1/500). After labelling, cells were washed and fixed in PBS containing 2% paraformaldehyde and stored at 4 °C before flow cytometry acquisition within 24 h.

### Flow cytometry analysis

All events (∼1,200,000–1,800,000 events per sample) were collected on a BD FACSymphony A5 Cell Analyzer (BD Biosciences). The lymphocytes were gated for further analysis, as described in Extended Data Fig. 2 using FlowJo version 9.9.6 (BD Biosciences).

### PhenoSense Monoclonal Antibody Assay

The PhenoSense Monoclonal Antibody Assay (Labcorp-Monogram Biosciences) is a phenotypic cell-based infectivity assay capable of assessing the susceptibility of pseudovirions bearing plasma or PMBC-derived HIV-1 envelope proteins to anti-envelope monoclonal antibodies. Populations of full-length envelope sequences, amplified from either plasma derived HIV RNA from viremic individuals or cell-associated HIV DNA from aviremic individuals, were cloned into an envelope expression vector. Envelope expression vector populations and a HIV genomic reporter vector, with env replaced by luciferase, were cotransfected into producer cells to generate luciferase reporter pseudovirions. Pseudovirions were then were tested for neutralization sensitivity to 3BNC117 or 10-1074 in an in vitro cell-based assay.

Clinical cut-off values for neutralization sensitivity have not been established for the PhenoSense Monoclonal Antibody Assay. In this study, the following exploratory values were used to define monoclonal antibody sensitivity: IC_90_ < 1 µg ml^−1^ and a maximum per cent inhibition equal to or > 98%.

### Single-genome amplification of reservoir *env* genes

Genomic DNA (gDNA) was isolated using Puregene Cell and Tissue Kit (Qiagen, catalogue no. 158388) according to the manufacturer’s instructions. DNA concentration was measured using Nanodrop. HIV-1 envelope was amplified from gDNA by single-genome amplification (SGA). To achieve a Poisson distribution, in which ≤30% of wells yield a PCR product, gDNA was endpoint diluted in 384-well plates. The first-round PCR was performed at 94 °C for 2 min; 94 °C for 15 s, 58.5 °C for 30 s, and 68 °C for 3 min × 35; and 68 °C for 15 min. One microlitre of the PCR product from first-round PCR was used as a template for second-round nested PCR at 94 °C for 2 min; 94 °C for 15 s, 61 °C for 30 s, and 68 °C for 3 min × 45; and 68 °C for 15 min. PCR2 products were checked using 1% 96-well E-Gels (Invitrogen). Bands with the expected size of the HIV-1 envelope were subjected to library preparation and sequencing using the Illumina Miseq platform (Control Software v3.1.0.13). All PCRs were performed using Platinum Taq Polymerase. Primers used in the first round were envB5out 5′–TAGAGCCCTGGAAGCATCCAGGAAG–3′ and envB3out 5′–TTGCTACTTGTGATTGCTCCATGT–3′. In cases where there was no amplification in the first round, 3′ half-genome primers were used instead (B3F3 5′-TGGAAAGGTGAAGGGGCAGTAGTAATAC-3′ and R3B6R 5′-TGAAGCACTCAAGGCAAGCTTTATTGAGGC-3′). The second-round nested primers used were envB5in 5′–TTAGGCATCTCCzTATGGCAGGAAGAAG–3′ and envB3in 5′–GTCTCGAGATACTGCTCCCACCC–3′.

### Q4PCR

Q4PCR was performed as previously described^21,39^. In brief, genomic DNA from 1 million to 5 million total CD4^+^ T cells was isolated using the Gentra Puregene cell kit (Qiagen) or phenol-chloroform, and the DNA concentration was measured using a Qubit high-sensitivity kit (Thermo Fisher Scientific). Next, an outer PCR (NFL1) was performed on genomic DNA at a single-copy dilution using outer PCR primers BLOuterF (5′-AAATCTCTAGCAGTGGCGCCCGAACAG-3′) and BLOuterR^37^ (5′-TGAGGGATCTCTAGTTACCAGAGTC-3′). Undiluted 1-µl aliquots of the NFL1 PCR product were subjected to a Q4PCR reaction using a combination of four primer–probe sets that target conserved regions in the HIV-1 genome. Each primer–probe set consists of a forward and reverse primer pair as well as a fluorescently labelled internal hydrolysis probe as previously described^21^ as follows: PS forward, 5′-TCTCTCGACGCAGGACTC-3′; reverse, 5′-TCTAGCCTCCGCTAGTCAAA-3′; probe, 5′-/Cy5/TTTGGCGTA/ TAO/CTCACCAGTCGCC-3′/IAbRQSp (Integrated DNA Technologies); env forward, 5′-AGTGGTGCAGAGAGAAAAAAGAGC-3′; reverse, 5′-GTCTGGCCTGTACCGTCAGC-3′; probe, 5′-/VIC/CCTTGGGTTCTTGGGA-3′/MGB (Thermo Fisher Scientific); gag forward, 5′-ATGTTTTCAGCATTATCAGAAGGA-3′; reverse, 5′- TGCTTGATGTCCCCCCACT-3′; probe, 5′-/6-FAM/CCACCCC- AC/ZEN/AAGATTTAAACACCATGCTAA-3′/ IABkFQ (Integrated DNA Technologies); and pol forward, 5′-GCA CTTTAAATTTTCCCATTAGTCCTA-3′; reverse, 5′-CAAATTTCTACTAATGCTTTTATTTTTTC-3′; probe, 5′-/NED/AAGCCAGGAATGGA-TGGCC-3′/ MGB (Thermo Fisher Scientific). Each Q4PCR reaction was performed in a 10-µl total reaction volume containing 5 µl TaqMan universal PCR master mix containing Rox (catalogue no. 4304437; Applied Biosystems), 1 µl diluted genomic DNA, nuclease-free water, and the following primer and probe concentrations: PS, 675 nM forward and reverse primers with 187.5 nM PS internal probe; env, 90 nM forward and reverse primers with 25 nM env internal probe; gag, 337.5 nM forward and reverse primers with 93.75 nM gag internal probe; and pol, 675 nM forward and reverse primers with 187.5 nM pol internal probe. Quantitative PCR (qPCR) conditions were 94 °C for 10 min, 40 cycles of 94 °C for 15 s, and 60 °C for 60 s. All qPCRs were performed in a 384-well plate format using the Applied Biosystem QuantStudio 6 or 7 Flex real-time PCR system. qPCR data analysis was performed as previously described^21^ using ThermoFisher Design and Analysis Software 2.4.3. Generally, samples showing reactivity with two or more of the four qPCR probes were selected for a nested PCR (NFL2) with the exception of participant 5108 for whom samples showing reactivity with one or more of the four qPCR probes were selected for NFL2. This focused approach detects the subset of defective proviruses that are positive for at least 2 of the 4 oligonucleotide probes which in turn leads to an underestimation of the absolute number of defective proviruses. The NFL2 reaction was performed on undiluted 1-µl aliquots of the NFL1 PCR product. Reactions were performed in a 20-µl reaction volume using Platinum Taq high-fidelity polymerase (Thermo Fisher Scientific) and PCR primers 275F^37^ (5′-ACAGGGACCTGAAAGCGAAAG-3′) and 280R (5′-CTAGTTACCAGAGTCACACAACAGACG-3′) at a concentration of 800 nM. Library preparation and sequencing were performed as previously described^21^. Sampling time points without recovery of intact sequences were determined at lower limit of detection for statistical analyses. Limit of detection is calculated as half of the proviral frequency assuming one intact sequence out of the highest input of sampled cells.

### Single-genome amplification of plasma rebound virus *env* genes

Sequencing of HIV-1 plasma rebound *env* genes was performed as previously described^12,51^.

### HIV-1 genome reconstruction

HIV-1 genome reconstruction was performed using our in-house pipeline, Defective and Intact HIV genome Assembler (DIHIVA), a pipeline for the assembly of raw sequencing reads into annotated HIV genomes, capable of reconstructing thousands of genomes within hours. First, it removes PCR amplification and performs error correction using clumpify.sh from BBtools package v38.72 (http://sourceforge.net/projects/bbmap). After, a quality-control check is carried out by Trim Galore package v0.6.4 (https://github.com/FelixKrueger/TrimGalore) to trim Illumina adapters and low-quality bases. The pipeline also uses bbduk.sh from BBtools package to remove possible contaminant reads using HIV sequences from the Los Alamos HIV database (https://www.hiv.lanl.gov) as a positive control. A k-mer-based assembler, SPAdes v3.13.0^52^ is used to assemble the HIV-1 sequences. The longest assembled contig is aligned via BLAST to HXB2 HIV-1 sequence to set the correct orientation and transfer annotations. Sequences with double-peaks, that is, regions indicating the presence of two or more viruses in the sample (cut-off consensus identity for any residue < 70%), or samples with a limited number of reads (≤500 sequencing reads) are omitted from downstream analyses. Finally, reconstructed sequences are classified as intact or defective proviruses. We divided defective proviral sequences into five subcategories: (1) non-functional, that is, complete, full-length genome however with one or multiple early stop codons in at least one coding genes; (2) major splice donor mutation mutation, that is, complete, full-length, in-frame genome with a mutation in the 5′ donor splice site 1 (D1); (3) missing internal genes, that is, truncated provirus, with large internal deletions spanning one or more of the 9 HIV coding genes; (4) structural variation, that is, genomes containing indels, duplications or inversions; (5) LTR defect, that is, genomes missing either 3′ LTR or 5′ LTR and PSI region

### Post hoc model for 10-1074 sensitivity testing

Reconstructed *env* sequences were analysed for the presence of computationally predicted 10-1074 escape mutations^53^. The *env* sequences were classified as sensitive when obeying all of the following amino acid rules (using HXB2 numbering): 332: N; 333: not P; 334: S or T; 325: D, N, or T and 330: H or Y. The performance of the model was tested against a 10-1074 neutralization panel that included 557 strains (*n* = 371 10-1074-sensitive (IC_50_ = 2.0 µg ml^−1^), *n* = 186 10-1074-resistant (IC_50_ ≥ 2.0 µg ml^−1^)). Of 393 strains predicted to be sensitive, 366 were shown to be true sensitive and 27 were false sensitive. Of 164 strains predicted to be resistant 159 were shown to be true resistant and 5 were false resistant resulting in a sensitivity of 99% and specificity of 85%.

### Phylogenetic analysis

Nucleotide alignments of *env* sequences were translation aligned using MAFFT v7.487 under the BLOSUM62 cost matrix. Maximum likelihood phylogenetic trees were then generated from these alignments with PhyML v3.1 using the general time-reversible (GTR) model with 1,000 bootstraps.

### Statistical analysis

Rates of viral rebound 12 weeks after ART interruption in group 1 and group 2 were calculated. Differences in time to viral rebound after ART interruption and after last antibody infusions between group 1 and a shorter antibody combination therapy study^12^ were determined using Kaplan–Meier curve comparison and log-rank (Mantel–Cox) tests. Differences in time to viral rebound between group 1 and group 2 were also determined. Differences in time since diagnosis and time on uninterrupted ART between bNAb therapy and ART-alone group were determined using two-tailed Mann–Whitney U-test. Differences in time to viral rebound between participants with phenotypically resistant, partially resistant or sensitive reservoir viruses was determined using one-way ANOVA with Tukey multiple comparison test. Differences in log normalized frequencies of intact and defective proviral genomes per 10^6^ CD4^+^ T cells were determined using paired Student’s *t*-test. Relative change in proviral frequencies was determined using two-tailed Wilcoxon matched-pairs signed-rank test for matched comparisons within bNAb therapy and ART-alone group. Pharmacokinetic parameters were estimated by performing a non-compartmental analysis using Phoenix WinNonlin Build 8.3 (Certara), using all available pharmacokinetic data starting with the time point after the last infusion of 10-1074 from the TZM-bl assay. These and all other statistical tests as indicated in the manuscript and figure legends were performed using GraphPad Prism 9.1.

### Data presentation

Figures were arranged in Adobe Illustrator 2021.

### Reporting summary

Further information on research design is available in the Nature Research Reporting Summary linked to this paper.

## Online content

Any methods, additional references, Nature Research reporting summaries, source data, extended data, supplementary information, acknowledgements, peer review information; details of author contributions and competing interests; and statements of data and code availability are available at 10.1038/s41586-022-04597-1.

### Supplementary information

Reporting Summary

Supplementary Table 1 Individual participant demographics and baseline clinical characteristics (bNAb therapy groups).

Supplementary Table 2 Individual participant demographics and baseline clinical characteristics (ART-alone group).

Supplementary Table 3 Adverse events reported during study follow-up.

Supplementary Table 4 HIV-1 RNA levels, CD4^+^ T cell counts and serum antibody levels (group 1).

Supplementary Table 5 HIV-1 RNA levels, CD4^+^ T cell counts and serum antibody levels (group 2).

Supplementary Table 6 PhenoSense results.

Supplementary Table 7 Genotypic sensitivity analysis.

Supplementary Table 8 Reservoir measurements.

## Figures and Tables

**Extended Data Table 1:**
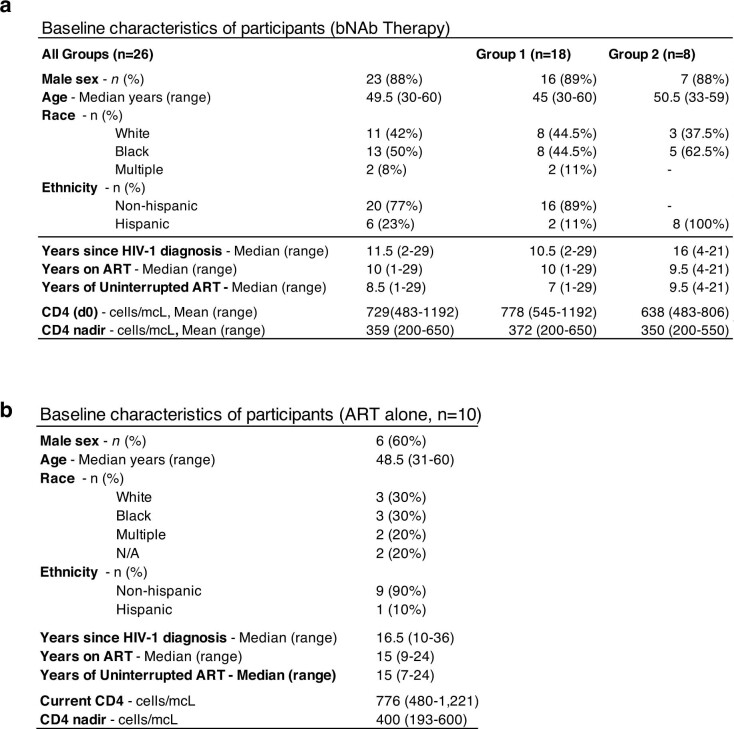
Study participant demographics

**a**, Table showing demographic characteristics of bNAb Therapy groups combined and Group 1 and 2 participants separately. **b**, Table showing demographic characteristics of ART alone group participants.

## Data Availability

All viral sequences have been deposited in GenBank with accession numbers OM203551 to OM209990, MT189273 to MT191008 and MW059111 to MW063083.

## Code availability

Computer code to process viral sequences is available at GitHub: https://github.com/stratust/DIHIVA.
