# Supplementary material for: Prolonged viral suppression with anti-HIV-1 antibody therapy
Source: Nature. 2022 Apr 13;606(7913):368–74. doi: 10.1038/s41586-022-04597-1 (PMC9177424; doi:10.1038/s41586-022-04597-1)
Supplement: Supplementary file 1 — Reporting Summary [file 41586_2022_4597_MOESM1_ESM.pdf]

nature research

Corresponding author(s): Marina Caskey and Michel C. Nussenzweig

Reporting Summary

Nature Research wishes to improve the reproducibility of the work that we publish. This form provides structure for consistency and transparency in reporting. For further information on Nature Research policies, see Authors & Referees and the Editorial Policy Checklist.

Statistical parameters

When statistical analyses are reported, confirm that the following items are present in the relevant location (e.g. figure legend, table legend, main text, or Methods section).

n/a	Confirmed	
☐	☒	The exact sample size (*n*) for each experimental group/condition, given as a discrete number and unit of measurement
☐	☒	An indication of whether measurements were taken from distinct samples or whether the same sample was measured repeatedly
☐	☒	The statistical test(s) used AND whether they are one- or two-sided<br>*Only common tests should be described solely by name; describe more complex techniques in the Methods section.*
☒	☐	A description of all covariates tested
☐	☒	A description of any assumptions or corrections, such as tests of normality and adjustment for multiple comparisons
☐	☒	A full description of the statistics including central tendency (e.g. means) or other basic estimates (e.g. regression coefficient) AND variation (e.g. standard deviation) or associated estimates of uncertainty (e.g. confidence intervals)
☐	☒	For null hypothesis testing, the test statistic (e.g. *F*, *t*, *r*) with confidence intervals, effect sizes, degrees of freedom and *P* value noted<br>*Give P values as exact values whenever suitable.*
☒	☐	For Bayesian analysis, information on the choice of priors and Markov chain Monte Carlo settings
☒	☐	For hierarchical and complex designs, identification of the appropriate level for tests and full reporting of outcomes
☐	☒	Estimates of effect sizes (e.g. Cohen's *d*, Pearson's *r*), indicating how they were calculated
☐	☒	Clearly defined error bars<br>*State explicitly what error bars represent (e.g. SD, SE, CI)*

Our web collection on statistics for biologists may be useful.

Software and code

Policy information about availability of computer code

Data collection	iRIS Version 11.02 by iMedRIS for clinical data collection and management; QuantStudio 6 and 7 Software v1.7.2 was used for qPCR assays; MiSeq Control Software v3.1.0.13 (Illumina), BD FACSymphony A5 Cell Analyzer (BD Biosciences) for Flow Cytometry data collection
Data analysis	Pharmacokinetics analysis was performed with Phoenix WinNonlin Build 8.3 (Certara), Flow Cytometry data analysis was performed using FlowJo version 9.9.6 (BD Biosciences).<br>ThermoFisher Design and Analysis Software 2.4.3 for qPCR analysis; GraphPad Prism 9.2; Microsoft Excel v16.54; Adobe Illustrator 2021 v25.3.1,<br>HIV-1 genome reconstruction was performed using our in-house pipeline, DIHIVA (Defective and Intact HIV genome Assembler, available at GitHub https://github.com/stratust/DIHIVA). PCR error correction uses clumpify.sh from BBtools package v38.72 (http://sourceforge.net/projects/bbmap). Quality-control check is carried out by Trim Galore package v0.6.4 (https://github.com/FelixKrueger/TrimGalore).

For manuscripts utilizing custom algorithms or software that are central to the research but not yet described in published literature, software must be made available to editors/reviewers upon request. We strongly encourage code deposition in a community repository (e.g. GitHub). See the Nature Research guidelines for submitting code & software for further information.

Data

Policy information about availability of data

All manuscripts must include a data availability statement. This statement should provide the following information, where applicable:

- Accession codes, unique identifiers, or web links for publicly available datasets
- A list of figures that have associated raw data
- A description of any restrictions on data availability

Data are provided in SI Tables 1-8. All viral sequences have been deposited in GenBank with the accession no. OM203551 to OM209990, MT189273 to MT191008, and MW059111 to MW063083.

Field-specific reporting

Please select the best fit for your research. If you are not sure, read the appropriate sections before making your selection.

☒ Life sciences ☐ Behavioural & social sciences ☐ Ecological, evolutionary & environmental sciences

For a reference copy of the document with all sections, see nature.com/authors/policies/ReportingSummary-flat.pdf

Life sciences study design

All studies must disclose on these points even when the disclosure is negative.

Sample size	With the expectation that at least 85% of people living with HIV will experience viral rebound by 12 weeks after discontinuing ART the upper confidence limit (UCL) of a 95% Clopper-Pearson confidence intervals was calculated for different sample sizes ranging from 5 to 30 and hypothetical true rates of viral rebounds. With a sample size of 18 (Group 1) and a true rate of rebound of 50% (or 10 rebounds before week 12 of ATI), there was an estimated probability of 86% of the UCL being below 85%. These probabilities can be seen as the power to reject the null hypothesis (H0= 85% rate of rebound after ART discontinuation) and accept the alternative hypothesis of a lower rate of rebound after the study interventions for a given sample size.
Data exclusions	No data were excluded.
Replication	This study was a clinical trial and the analyses were performed on individual trial participants. Experiments did not include replicates as all participants and data points are unique. All available data is included in the manuscript.
Randomization	Study participants were enrolled sequentially according to eligibility criteria and randomized to either Group 1 or Group 2 in a 3:1 ratio.
Blinding	The study was open label.

Reporting for specific materials, systems and methods

Materials & experimental systems

n/a	Involved in the study
☐	☒ Unique biological materials
☐	☒ Antibodies
☒	☐ Eukaryotic cell lines
☒	☐ Palaeontology
☒	☐ Animals and other organisms
☐	☒ Human research participants

Methods

n/a	Involved in the study
☒	☐ ChIP-seq
☐	☒ Flow cytometry
☒	☐ MRI-based neuroimaging

Unique biological materials

Policy information about availability of materials

Obtaining unique materials	The study included the analyses of blood samples collected from enrolled participants living with HIV. These samples were obtained under an IRB approved protocol for the purposes of this study and associated analytical plan.

Antibodies

Antibodies used

3BNC117 and 10-1074 are investigational anti-HIV-1 neutralizing antibodies manufactured for clinical use. They are being investigated under US FDA INDs 118225 and 123713.
Antibodies used for phenotypic characterization and surface staining by Flow Cytometry were:

BUV395 Mouse Anti-Human CD3 Clone SK7 – BD # 564001 Dilution 1/100
BUV805 Mouse Anti-Human CD4 Clone OKT4 – BD # 750976 Dilution 1/200
BV480 Mouse Anti-Human CD8 Clone RPA-T8 – BD # 566121 Dilution 1/500
APC-Cy7 Mouse Anti-Human CD19 Clone SJ25C1 – BD # 557791 Dilution 1/250
APC-H7 Mouse Anti-Human CD20 Clone 2H7 – BD # 560734 Dilution 1/100
APC/Cy7 anti-human CD66b Antibody Clone G10F5 – Biolegend # 305126 Dilution 1/250
APC-H7 Mouse Anti-Human CD14 Clone M5E2 – BD # 561384 Dilution 1/150
BB790 Mouse Anti-Human CD38 Clone HIT2 – BD # 624296 Dilution 1/250
BUV737 Mouse Anti-Human CD69 Clone FN50 – BD # 564439 Dilution 1/500
BV750 Mouse Anti-Human CD71 Clone M-A712 – BD # 747308 Dilution 1/100
BUV615 Mouse Anti-Human CD25 Clone 2A3 – BD # 564439 Dilution 1/300
BB630 Mouse Anti-Human CD152 Clone BNI3 – BD # 624294 Dilution 1/250
BV421 Mouse Anti-Human CD279 (PD-1) Clone EH12.1 – BD # 562516 Dilution 1/500
BB660 Mouse Anti-Human TIGIT Clone 741182 – BD # 624295 Dilution 1/500
PE Mouse Anti-Human LAG-3 (CD223) Clone T47-530 – BD # 565616 Dilution 1/250
BUV661 Mouse Anti-Human TIM-3 (CD366) Clone 7D3 – BD # 624285 Dilution 1/500
Viability reagent BD Horizon Fixable Viability Stain780 – BD # 565388 Dilution 1/1000

Validation

The amounts of antibody needed per staining are determined by titration experiments done before performing the procedure. The optimal concentration of an antibody is determined by a titration experiment in which typically 2 × 106 cells in a 20-µl volume are stained with 1×, 2×, 4× and 8× the amount of antibody. The optimal concentration for a specific antibody is the amount giving the best fluorochrome signal and the least fluorochrome spreading. To prepare the antibody staining solution, the titrated amount of antibody for all antibodies is mixed and made up to 20 µl with staining buffer. The staining volume is 40 µl total (20 µl of cell suspension + 20 µl of antibody staining solution). The staining volume and the amount of antibody should be scaled up according to the number of cells. The staining buffer contain 50% of PBS 1% FBS (vol/vol) and 50% of BD Horizon™ Brilliant Stain Buffer (BD # 566349).

Human research participants

Policy information about studies involving human research participants

Population characteristics

Eligible participants were adults aged 18–65 years, HIV-1-infected, on ART with plasma HIV-1 RNA levels of < 50 copies/ml for at least 12 month (one viral blip of >50 but <500 copies per ml during this period was allowed), plasma HIV-1 RNA levels < 20 copies/ml at screening visit, reported or confirmed CD4+ T cell count nadir of > 200 cells/µl and a current CD4+ T cell counts > 500 cells/µl. Participants on an ART regimen that included a non-nucleoside reverse transcriptase inhibitor (NNRTI) were switched to an integrase inhibitor-based regimen (dolutegravir plus tenofovir disoproxil fumarate and emtricitabine) at least four weeks before treatment interruption due to the prolonged half-life of NNRTIs. Exclusion criteria included medical history of resistance to 2 or more classes of antiretroviral medication, prior anti-HIV monoclonal antibody therapy, concomitant hepatitis B or C infection, clinically relevant physical findings, medical conditions or laboratory abnormalities and pregnancy or lactation. We enrolled 26 participants (23 male, 3 female) with a median age of 49.5 years in the bNAb Therapy group. We enrolled an additional 10 participants (6 male, 4 female) with a median age of 48.5 years in the ART alone group.

Recruitment

Study participants were recruited at the Rockefeller University Hospital, New York, USA, and the Massachusetts General Hospital, Boston, USA. Potential study participants responded to online or print advertisements or were referred for screening by primary care providers. Study participants were enrolled sequentially according to eligibility criteria and randomized to either Group 1 or Group 2 in a 3:1 ratio. All participants provided written informed consent before participation in the study and the study was conducted in accordance with Good Clinical Practice. The protocol was approved by the Food and Drug Administration in the USA and the Rockefeller University and Mass General Brigham Human Research Institutional Review Boards (IRBs). PBMC samples from 4 study participants in a parallel NIH/NIAID-sponsored clinical trial who received 3BNC117 in combination with 10-1074 during ART interruption (http://www.clinicaltrials. gov; NCT03571204) were included for reservoir assessments. The protocol was approved by the Institutional Review Board of the National Institute of Allergy and Infectious Diseases, National Institutes of Health.

Flow Cytometry

Plots

Confirm that:

☒ The axis labels state the marker and fluorochrome used (e.g. CD4-FITC).

☒ The axis scales are clearly visible. Include numbers along axes only for bottom left plot of group (a 'group' is an analysis of identical markers).

☒ All plots are contour plots with outliers or pseudocolor plots.

☒ A numerical value for number of cells or percentage (with statistics) is provided.

Methodology

Sample preparation	Frozen PBMCs were thawed and washed with PBS. Cells were then surface stained for 30 min in the dark at 4°C with viability reagent (BD Horizon Fixable Viability Stain; BD Biosciences) and a 13-color cocktail of mAbs containing surface antibodies against CD19 (Clone SJ25C1), CD20 (Clone 2H7), CD66b (Clone G10F5), CD14 (Clone M5E2), CD3 (Clone SK7), CD4 (Clone OKT4), CD8 (Clone RPA-T8), CD38 (Clone HIT2), CD69 (Clone FN50), CD71 (Clone M-A712), CD25 (Clone 2A3), CD152 (Clone BNI3), CD279 (Clone EH12.1), TIGIT (Clone 741182), CD223 (Clone T47-530), and CD336 (Clone 7D3). After labeling, cells were washed and fixed in PBS containing 2% paraformaldehyde and stored at 4°C before flow cytometry acquisition within 24 h.
Instrument	All events (~1,200,000–1,800,000 events per sample) were collected on a BD FACSymphony A5 Cell Analyzer (BD Biosciences).
Software	FlowJo version 9.9.6 (BD Biosciences) was used to analyze flow cytometry data.
Cell population abundance	Gating strategy used to define total CD4+ and CD8+ T cells. Dump channel includes CD19, CD20, CD14 and CD66b as well as Live/Dead. CD4+ T cells are identified as Dump−CD14−CD3+CD4+, whereas CD8+ T cells are Dump−CD14−CD3+CD8+.
Gating strategy	ThTotal CD4+ (blue) and CD8+ (red) T cells are identified by first gating on PBMCs (FSC-A versus SSC-A), and then on singlets (SSC-A versus SSC-W) and Dump- cells (CD19, CD20, CD14, CD66b and Viability Stain780 versus SSC-A). CD4+ T cells are identified as live Dump−CD14−CD3+CD4+, whereas CD8+ T cells are live Dump−CD14−CD3+CD8+. Positive expression of CD38, CD69, CD71, CD25, CD152, CD279, TIGIT, CD223 and CD366 by CD4+ T cells and CD8+ T cells is defined by FMOCs gates.

☒ Tick this box to confirm that a figure exemplifying the gating strategy is provided in the Supplementary Information.
